# Rapid evolutionary responses of life history traits to different experimentally-induced pollutions in *Caenorhabditis elegans*

**DOI:** 10.1186/s12862-014-0252-6

**Published:** 2014-12-10

**Authors:** Morgan Dutilleul, Jean-Marc Bonzom, Catherine Lecomte, Benoit Goussen, Fabrice Daian, Simon Galas, Denis Réale

**Affiliations:** Département des Sciences Biologiques, Université du Québec À Montréal, Montreal, Canada; Institut de Radioprotection et de Sûreté Nucléaire (IRSN), PRP-ENV/SERIS/LECO, Cadarache, Bât 183, BP 3, 13115 St Paul-lez-Durance, France; Université de Montpellier 1, Faculté de pharmacie, Laboratoire de Toxicologie, BP 14491, F-34093 Montpellier Cedex 5, France; Unit “Models for ecotoxicology and toxicology” (METO) INERIS Parc ALATA, BP2 60550 Verneuil-en-Halatte, France; Institut de Biologie du Développement de Marseille-Luminy, UMR7288, CNRS, F-13288 Marseille Cedex 9, France

**Keywords:** Experimental evolution, Phenotypic (co)variance, Local adaptation, Evolution of generalism, Pollutant

## Abstract

**Background:**

Anthropogenic disturbances can lead to intense selection pressures on traits and very rapid evolutionary changes. Evolutionary responses to environmental changes, in turn, reflect changes in the genetic structure of the traits, accompanied by a reduction of evolutionary potential of the populations under selection. Assessing the effects of pollutants on the evolutionary responses and on the genetic structure of populations is thus important to understanding the mechanisms that entail specialization to novel environmental conditions or resistance to novel stressors.

**Results:**

Using an experimental evolution approach we exposed *Caenorhabditis elegans* populations to uranium, salt and alternating uranium-salt environments over 22 generations. We analyzed the changes in the average values of life history traits and the consequences at the demographic level in these populations. We also estimated the phenotypic and genetic (co)variance structure of these traits at different generations. Compared to populations in salt, populations in uranium showed a reduction of the stability of their trait structure and a higher capacity to respond by acclimation. However, the evolutionary responses of traits were generally lower for uranium compared to salt treatment; and the evolutionary responses to the alternating uranium–salt environment were between those of constant environments. Consequently, at the end of the experiment, the population rate of increase was higher in uranium than in salt and intermediate in the alternating environment.

**Conclusions:**

Our multigenerational experiment confirmed that rapid adaptation to different polluted environments may involve different evolutionary responses resulting in demographic consequences. These changes are partly explained by the effects of the pollutants on the genetic (co)variance structure of traits and the capacity of acclimation to novel conditions. Finally, our results in the alternating environment may confirm the selection of a generalist type in this environment.

**Electronic supplementary material:**

The online version of this article (doi:10.1186/s12862-014-0252-6) contains supplementary material, which is available to authorized users.

## Background

During the last few decades numerous studies have highlighted the important role of anthropogenic disturbances on the occurrence and speed of contemporary evolution in wild populations (reviewed by [1-4]). Furthermore, evolution experiments have shown that fitness of populations subjected to heavy metals or pesticides can rebound within less than ten generations (e.g. [5-7]). These results indicate that – at least in these conditions – populations can show quick evolutionary responses to anthropogenically-induced selection pressures. However, when new environmental conditions appear, they can affect life history traits and thus may have major consequences for the demography of the populations. These demographic changes may in turn reduce the evolutionary potential of populations through increased genetic drift and reduced genetic variance for the traits under selection [8]. It is therefore important to evaluate the demographic consequences of a novel environment as they provide information on the potential for evolutionary rescue [8,9].

Several factors can reduce the evolutionary potential of a population facing novel environmental conditions. An organism can be viewed as an integrated system with functional, developmental and genetic associations among its different traits [10]. It is therefore important to consider the multivariate feature of traits in an organism to provide more robust predictions of the evolutionary trajectory of populations as a result of novel selection pressures [11]. The evolutionary potential of traits is constrained by the magnitude and sign of the genetic associations among the traits [12]. These associations are represented by the matrix **G** of additive genetic variance and covariance [13]. Consequently changes of **G**-matrix structure could modify the evolutionary trajectory of a population [14,15]. **G**-matrices are assumed to be highly stable over time [16,17]. Several studies, however, have shown that they can be easily altered (e.g. [15,18,19]), including by quick changes in environmental conditions [20,21].

When submitted to a new stressor, a population can change its evolutionary trajectory, which may lead to improved adaptive responses to the stressor. For instance, selection induced by pollution can favor genotypes allocating more resources to detoxification mechanisms [22,23]. To date, however, we are limited in our ability to predict which mechanisms and traits will be involved in response to the type of pollutant and its concentration, and if these responses can be generalized to all species. To improve our ability to predict the evolutionary patterns involved in response to different pollutants we need to compare the evolutionary responses of replicates of the same population of origin, subjected to different sources of pollution.

In addition, selection induced by pollutants is assumed to be directional, continuous and strong [3,24]. However, in a heterogeneous environment, antagonistic selection pressures induced by ecological factors other than the pollutant can both prevent the rapid evolution of a population in response to the pollutant [25,26] and maintain its genetic variation and therefore its evolutionary potential in response to potential new stressors (theoretical studies: [27-29]; empirical studies: [26,30,31]). Furthermore, temporally fluctuating environments seem to favor a generalist rather than a specialist way of life [32-35]. Populations that evolved in the presence of alternating stressors may thus cope less well with each stressor, and their evolutionary response may be slower than for populations that evolved in response to a single stressor.

In this study we used experimental populations of *Caenorhabditis elegans* to evaluate the evolution of life history traits in response to two different pollutants, salt (NaCl) and depleted uranium (U) – the radiological effects of exposure to depleted U are assumed to be neglected compared to the chemical effects [36]. We analyzed multivariate evolutionary responses to each of these pollutants and to an alternation of these pollutants. Our approach allowed us to test whether (i) the degree of evolutionary response to selection and the evolutionary patterns of life history traits differed according to the pollutant, (ii) the stability of environmental conditions or the alternating presence of the two pollutants affected the evolution of the traits differently, (iii) the regime of selection caused by each treatment affected the demography differently, and (iv) the temporal changes in the phenotypic/genetic (co)variance structure of life history trait differed according to the polluted environments.

## Results

### Evolutionary responses to different polluted environments

For hermaphrodites and males, trait changes across generations (i.e. slopes) differed according to the treatment. For traits measured in hermaphrodites, the model with the lowest deviance information criterion (DIC) included an interaction between treatment and generation, and covariance between traits (Table 1). We found similar results when we limited the analysis to each pair of traits, except between growth and late fertility [see Additional file 1]. For males the selected model included the interaction between treatment and generation, but including trait covariance did not significantly improve the fit of the model (Table 1). In both cases the replicate effects explained < 4% of the (co)variance among traits.

**Table 1:** **Comparison of multivariate mixed models including different effects**

**Effects included within the model**	**DIC**	Δ**DIC**
For her maphrodite traits		
-	−2506.140	-
Environment	−2538.996	−32.853
Environment + generation	−2588.716	−49.720
Environment × generation	**−2653.526**	**−64.810**
Environment × generation (no covariance)	445.699	3098.535
For male traits		
-	701.020	-
Environment	683.189	−17.831
Environment + generation	631.832	−51.357
Environment × generation	605.585	−26.247
Environment × generation (no covariance)	**608.146**	**2.561**

Effect of generation and environment (control, uranium, salt and alternating uranium-salt) on hermaphrodite (i.e. growth and total, early and late fertility) and on male (i.e. growth and body bend) traits, measured between generations 4 and 22 of the multigenerational experiment. We used multivariate mixed models with all the traits included as dependent variables, and compared different models using deviance information criterion (DIC). Left-hand side: characteristics of the fixed effects included in each model (the first DIC value corresponds to a simple model including only replicates as a random effect). Right-hand side: DIC of the model followed by the change (Δ) in DIC value between this model and the previous model that did not include the fixed effect. Except for the models shown at the last line for each sex, covariance between traits was allowed in the priors. In bold: selected models for which ΔDIC > 5, i.e. the model including interaction had a smaller DIC. Replicate effects in these models represent 3.3% and 2.8% of the total variance for traits measured in hermaphrodites and males, respectively.

**Figure 1:**
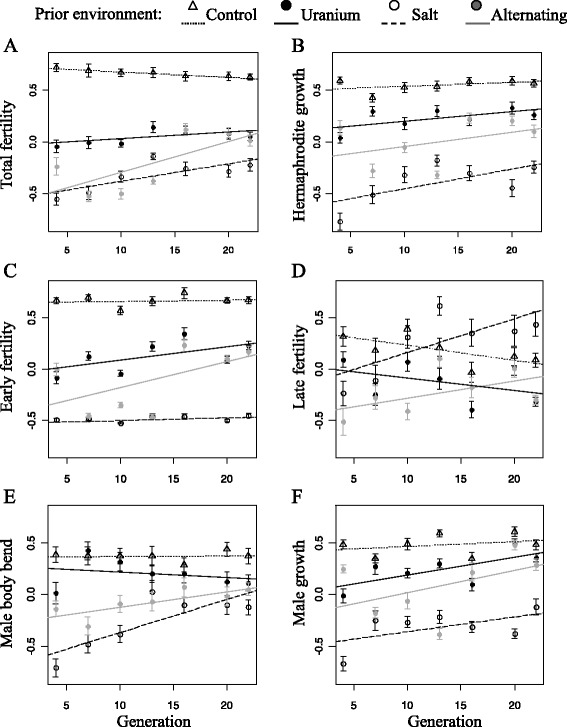
**Evolutionary responses of traits between generations 4 and 22.** Measures of hermaphrodite total fertility **(A)**, growth **(B)**, early **(C)** and late **(D)** fertility, and male growth **(E)** and body bend **(F)**. Each symbol corresponds to the mean value of the trait and its standard error (n = 18 individuals/treatment/generation). Trait values were rescaled prior to analysis by subtracting each value by the mean of the sample (i.e. all data for a trait across generations and treatments) and dividing it by twice the standard deviation. Control (empty triangles), uranium (filled black dots), salt (empty dots) and alternating uranium-salt (filled gray dots) environments. Regression lines correspond to posterior mode of the distribution for intercept and slope (generation was a continuous fixed effect). Small dashed line: control; black line: uranium; large dashed line: salt; gray line: alternating uranium-salt environments.

At generation 4, intercepts of traits were lower in polluted environments than in controls, and lower in the salt than uranium populations (although non-significant for late fertility in the latter case; Figure 1 [and see Additional file 2]). As shown by the 95% highest posterior density interval (HPDI) [see Additional file 2], traits did not change across generations in the control treatment, except for a slight reduction in late fertility (Figure 1). Evolutionary responses between generations 4 and 22 were generally higher to salt (1%–5% per generation) than uranium treatment (1%–2% per generation), and the strongest evolutionary response of fertility was to the selection imposed by the alternating treatment (Figure 1). The evolutionary responses of early fertility to uranium treatment, of late fertility to salt treatment and of both traits to the alternating treatment were significant (Figure 1C and D). Traits related to reproduction showed stronger evolutionary responses (2%–5% per generation) than traits related to growth (1%–2% per generation; Figure 1B and F). Male body bend increased between generations 4 and 22 for salt and alternating treatments, but not for uranium or controls (Figure 1E).

### Demographic consequences of evolution in different environments

**Figure 2:**
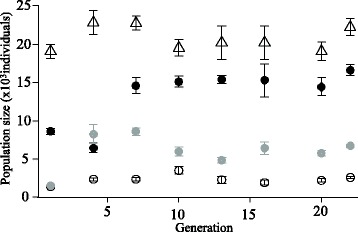
**Changes in average population size in the different treatments between generations 1 and 22.** Symbols show the mean value and standard error over six replicated populations in control (empty triangles), uranium (filled black dots), salt (empty dots) and alternating uranium-salt (filled gray dots) treatments.

For all treatments we started each replicate population with 500 individuals. After 3 days, estimated population size in the control environment reached 20 000 individuals on average, and fluctuated around this value over the 22 subsequent generations (Figure 2 [and see Additional file 3 for statistical analyses]). In the uranium treatment, estimated population size reached 9000 and 6000 individuals for generations 1 and 4, respectively. It then increased to reach a plateau at around 15 000 individuals during generations 7–22. In the salt treatment the estimated population size first decreased to < 1500 individuals during the first generation; it stayed low and varied around 3000 individuals during the whole experiment. In the alternating treatment, estimated population size started at around 1500 individuals during generation 1, and then fluctuated within 5000–9000 individuals, without any notable temporal trend.

### Comparison of (co)variance matrices

We did not find any covariance between growth and body bend in males. Furthermore, tests for traits in males revealed angles between the first eigenvector of each combination of two matrices of 0° in all environments (data not shown). We thus only present matrix comparisons for hermaphrodites.

**Figure 3:**
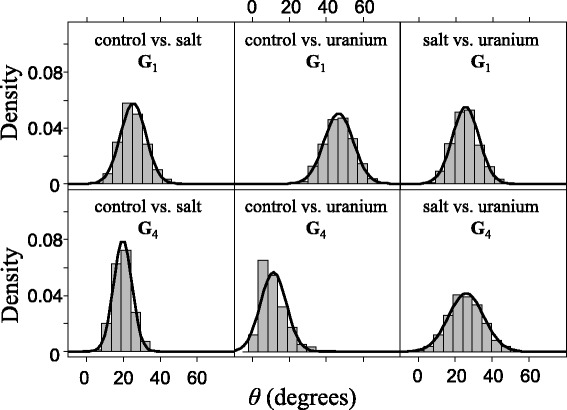
**Angle between different environments.** Histogram and density curve of the distribution of the angle (*θ*) between different environments. *θ* obtained by a resampling procedure, between the first principal component (eigenvector) of both matrices of (co)variance for traits measured in hermaphrodites (total, early and late fertility, and growth) between populations from two environments: control vs. salt, control vs. uranium, and salt vs. uranium. We used this procedure for the periods **G**_1_ (generations 1–4) and **G**_4_ (generations 4–22). Error bars denote 95% confidence intervals for *θ* obtained using a resampling procedure.

We found a strong divergence of **G**_1_ matrices (see description of **G**_1_, **G**_2_, **G**_3_ and **G**_4_ in Material and methods) between control, uranium and salt treatments (Figure 3 top; 24° < *θ* < 47°, and in all cases CIs > 0). For **G**_4_ matrices divergence was moderate between salt and both control and uranium treatments (Figure 3 bottom; 19° < *θ* < 26°, and in all cases CIs > 0). Divergence in **G**_4_ matrices was non-significant between uranium and control treatments (*θ* < 11°, and CI ≈ 0).

**Figure 4:**
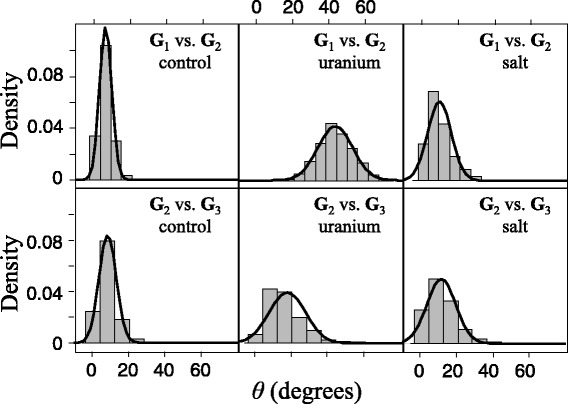
**Angle between different periods.** Histogram and density curve of the distribution of the angle (*θ*) between different periods. *θ* obtained by a resampling procedure, between the first principal component (eigenvector) of both matrices of (co)variance for traits measured in hermaphrodites (total, early and late fertility, and growth) between two periods **G**_1_ (generations 1–4) vs. **G**_2_ (generations 4–10); and **G**_2_ vs. **G**_3_ (generations 13–22). We used this procedure for the populations from control, uranium and salt environments. Error bars denote 95% confidence intervals for *θ* obtained using a resampling procedure.

**G**_1_ and **G**_2_ matrices did not differ in the control environment (Figure 4 top; *θ* = 3°, and CI ≈ 0). We found a strong divergence between these matrices in the uranium treatment (*θ* = 43°, and CI > 0), and a weak divergence in the salt treatment (*θ* = 5°, and CI > 0). Divergence between **G**_2_ and **G**_3_ was negligible in the three environments (Figure 4 bottom; *θ* < 9°, and CIs ≈ 0), indicating that all matrices were more stable during that period.

**Figure 5:**
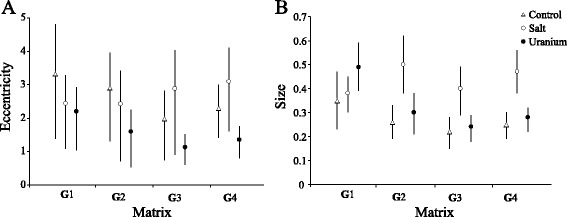
**Matrix eccentricity and matrix size.** Measures of matrix eccentricity **(A)** and size **(B)** of (co)variance matrices for traits measured in hermaphrodites in the control (empty triangles), the uranium (filled black dots) and the salt (empty dots) environments. Error bars represent 95% highest and lowest of confidence intervals. We used **G**_1_ (generations 1–4), **G**_2_ (generations 4–10), **G**_3_ (generations 13–22) and **G**_4_ (generations 4–22) matrices.

Eccentricity and size of the **G**_1_ matrices did not differ significantly between the environments (Figure 5). Eccentricity decreased significantly across the subsequent matrices in the uranium populations, indicating a decrease in stability of the correlation between traits in this polluted environment (Figure 5A; CI for the subtractions of distributions of eccentricity between **G**_1_ and **G**_2_ did not overlap 0). There was a significant decline in matrix size for control and uranium compared to the salt environments at **G**_4_ (Figure 5B; CIs for the subtractions of distributions of size between **G**_4,salt_ and **G**_4,control_ or **G**_4,uranium_ did not overlap 0), indicating a decrease in the overall (co)variance of traits in these two environments. However, matrix size appeared stable over time for the control (CIs for the subtraction of distributions of size between **G**_1_ and **G**_2_ and between **G**_2_ and **G**_3_ overlapped 0) unlike the uranium environment (CI for the subtraction of distributions of size between **G**_1_ and **G**_2_ did not overlap 0). The two pollutants affected eccentricity and size differently: uranium decreased both eccentricity and size, whereas salt resulted in both parameters remaining stable over time (Figure 5A, B).

## Discussion

This laboratory study demonstrated that *C. elegans* populations can evolve towards a higher resistance to pollutants in only a few generations. Overall, we found a stronger evolutionary response of populations to salt than uranium treatment (shown by the stronger initial decrease in life history traits and by the greater slopes for salt across generations). However, the results suggest that pollutants drove the evolution of populations towards different life histories: salt populations slowed down their life histories by producing more eggs during late fertility (i.e. positive evolution of late fertility), whereas uranium populations showed faster life histories by producing more eggs during early fertility (i.e. positive evolution of early fertility). Changes in life history features in the presence of different pollutants were also revealed by demographic changes; at the end of the experiment the rates of increase were similar for uranium and controls, but were much lower for salt and alternating treatments. Furthermore, evolution in the alternating pollutant regime was intermediate between the two individual treatments. Towards the end of the experiments we detected changes in the (co)variance structure of traits in uranium and salt compared to control populations, and compared to different periods for the same treatment. These results suggest that trait (co)variance matrices were not always stable in a changing environment, and changed according to the novel conditions. Overall, salt seemed to maintain the features of the matrix (stable eccentricity and size) through time, whereas uranium populations showed a decrease in trait variance (i.e. matrix size) and covariance (i.e. matrix eccentricity and size).

### Microevolutionary trajectories in response to different pollutants

In the presence of uranium or salt the *C. elegans* populations clearly showed evidence of stress, as their life history traits and Darwinian fitness (i.e. rate of increase *sensu* [37]) were all strongly affected. Our results from generation 4 onward suggest that most phenotypic changes during the experiment were caused by cross-generation genetic changes in response to novel selection pressures [3,24]. The large number of individuals (500) used to seed new Petri plates at each generation and the low variance between replicate populations within a treatment (< 4% of the total trait variance), showed an absence of random divergence between replicates – this rules out the possibility that genetic drift could be responsible for the observed long-term changes across generations. Similarly the absence of trends in control populations suggests that uncontrolled environmental conditions had negligible effects on changes in traits measured during the experiment. However, during the first four generations the effects of selection may have been obscured by potential effects such as intragenerational or transgenerational (i.e. parental effects) phenotypic plasticity (e.g. [38]). Intragenerational and transgenerational effects, however, were unlikely to be responsible for cross-generation changes observed from generation 4 onward, i.e. once the animals experienced a stable environment the parental effects did not generate any new variation among individuals caused by the novel environment [38]. Therefore, after generation 4, changes at the phenotypic level probably reflect microevolutionary (i.e. genetic) response to selection. These microevolutionary changes occurring in the different polluted environments confirm the previous findings of Lopes *et al.* [39] on the capacity of a genetically diverse population of *C. elegans* to respond to selection by a pollutant pesticide. Epigenetic effects could also be responsible for some evolutionary changes throughout the experiment [40,41]. Epigenetic effects might have played a role in evolutionary changes in this experiment if they generated epigenetic inheritance responsible for some phenotypic variation similar to genetic inheritance [42-44]. Whatever source of variation is the origin of these changes, *C. elegans* populations have the potential to rapidly change their traits in the presence of novel pollutants, resulting in rapid improvement of fitness across generations.

### Environment-dependent evolutionary divergence

Despite a strong and immediate impact, in a few generations, populations quickly responded to both pollutants. Pollutants had dramatic effects on life history traits, and these effects differed with the type of pollutant.

Within the first three generations, in the presence of a pollutant, populations reacted with a strong decrease in early fertility and growth, and to a lesser extent in late fertility [see Additional file 4]. Furthermore, populations subjected to salt and to alternating pollutants showed a strong decrease in early survival; however, for uranium the survival remained similar to control populations [see Additional file 5]. These differences between environments reflect different selection pressures imposed by pollutants on traits, indicating that several mechanisms may be involded in these short-term responses. Further experiments with different stress and control treatments in a large dataset of individual traits, in the first three generations of exposure, are required to address the mechanisms involved in these responses. A comparison between generations or treatments would allow discrimination and quantification of the role of each mechanism.

After generation 4, populations reacted by increasing their total fertility. However, salt and uranium treatments affected fertility at different life stages. For salt, fertility increased later but not early in life (Figure 1 [and see Additional file 2]); furthermore, salt favored hermaphrodite growth and stronger body bend frequency. In contrast, for uranium, fertility increased early in life but did not change later; also, uranium did not affect hermaphrodite growth and male locomotion. At this stage it is too speculative to infer why the different pollutants led to the evolution of different life histories, but our results highlight the need for more studies on the divergent effects of pollutants on life history.

Growth for hermaphrodites and males in all polluted environments improved over time compared to controls, except for hermaphrodites in uranium. In some cases, we were unable to clearly conclude that the evolutionary response was significant. However, given the observed trends, the evolutionary response could have become significant if the experiment was performed for a few more generations. Another explanation could be that fitness is more strongly related to fertility than to growth [45] and thus that growth may be subject to weaker selection pressures and not evolve as quickly as fertility.

Locomotion was not affected by uranium after generation 4. In contrast there was an evolutionary response to salt and alternating treatments after a reduction in initial generations. Pollutants commonly decrease the frequency of body bends in the short term (e.g. [46]). Since locomotion behavior promotes encounter rate between males and hermaphrodites [39,47,48], outcrossing could be affected in polluted environments. It should be noted that compared to self-fertilization outcrossing permits hermaphrodites to double or quadruple their fertility [49,50]. However, the ratio of males, also an index of conservation of outcrossing rate [51-53], was only slightly affected in the uranium and the alternating environments, and was not affected in the salt environment [see Additional file 5]. The effects of salt on locomotion, associated with a reduction in survival, could partly explain the lower intrinsic population growth in this environment.

Evolution in the alternating uranium-salt treatment was intermediate between the two individual treatments – similar to evolution for salt at the beginning of the experiment and became more similar to that for uranium at the end. Temporal fluctuating regimes can lead to antagonistic selection pressures and thus to slower evolutionary responses (e.g. alternating light–dark in *Chlamydomonas* algae; [32]). The intermediate response in the salt-uranium alternating treatment may support this hypothesis. An intermediate evolutionary trajectory may also reflect a cost of lost alleles due to selection caused by the recurrent change of phenotypic optima (see [54]). In such a case, it appears that the change in phenotypic optima was not completely symmetrical and that, compared to salt, selection in uranium had a greater effect on the evolutionary responses of these populations. Previous studies have found cases of adaptation to heterogeneous environmental conditions through evolution towards a more generalist way of life [32,34,35]. In these cases, the process involved in the evolution of generalism seems to be mutation accumulation [55]. The present study involved only 22 generations, and mutation rate was likely insufficient to be the cause of the evolutionary changes observed [56,57].

### Demographic consequences

None of the populations subjected to pollutants reached the rate of increase of the control populations during the study period. Therefore, despite rapid evolutionary changes, populations were unable to revert to their original fitness level unless these changes modified their fitness optimum. At the end of the experiment, the population rate of increase for uranium treatment reached about 75% of the control rate – in contrast it stabilized at around 35% for the alternating treatments and < 15% in the salt treatments.

Although the duration of our experiment was not long enough to estimate the potential for evolutionary rescue, it should be noted that all the populations could maintain a positive rate of increase [8]. Furthermore, the impact of the different pollutants on life histories and their demographic consequences suggest that different selection pressures leading to different adaptive processes may also generate different probabilities for evolutionary rescue. Evolutionary rescue depends on factors such as initial genetic diversity, population size, the intensity of selection imposed by the environment and the evolutionary history of the population [8,9,58]. Our results suggest that another factor affecting the potential for evolutionary rescue is the type of life history evolution consequent to new selection pressures imposed by the stress: uranium quickly allowed the populations to grow to a level similar to the control populations. In contrast, by slowing the pace of life of populations, salt may lower the potential for evolutionary rescue.

### Changes in P- and G-matrices and environmental conditions

The first eigenvector of a **G**-matrix represents the line of least genetic resistance of a population, and thus **G** can be used to predict the evolutionary potential and trajectory of a population [11]. Therefore changes in the direction of that eigenvector across generations, or differences between environmental conditions, provide information on the evolvability of a population in these conditions, and on the effect of the novel environment on evolutionary trajectory. Furthermore, the stability of the **G**-matrix is an important assumption for predicting the evolutionary constraints imposed by the genetic structure of a population in a novel environment [13,59].

Quantitative genetic studies historically assumed that the **G**-matrix was stable through time and conditions, as this assumption enabled researchers to predict evolutionary changes from the action of selection on the matrix [16,17,19]. However, recent studies have shown that this was not the case [14,15,18,20,21]. In support of these previous findings, our study showed very rapid changes in the **P-** and **G**-matrix structure in *C. elegans* populations subjected to novel environmental conditions. Studies have revealed divergence between **G**-matrices, after hundreds or thousands of generations of evolution in natural populations, as a result of combined selection, drift and mutational effects (e.g. [15,18,60,61]). **G**-matrices can also change within a few generations as a result of rapid changes in the adaptive landscape [16]. Sgrò and Blows [20] showed some alteration of the genetic structure in *Drosophila* populations that evolved for 30 generations in different heat stress environments. Finally, as our results show, **G** can change within a few generations, before mutations or potential selection could have affected it. **G**-matrix instability has already been shown on a very short term (e.g. within a generation by [21]). Furthermore, short-term changes in estimates of genetic variance and covariance have been found as a result of environmental changes [62,63].

We also observed divergence in the **P**- and **G**-matrices for populations that experienced different environmental conditions. For uranium the most important changes occurred within the first four generations (Figures 3 and 4). Furthermore, matrix eccentricity and size decreased in the uranium populations, revealing a decrease in trait variance and covariance in this environment. Changes in the orientation of the matrix, and in matrix eccentricity and size, within the first four generations followed by stability after generation 4 for uranium suggest that in this novel environment epigenetic or acclimation effects may have caused changes in the **P**-matrix. In contrast, divergence in matrix orientation between salt and the other populations was moderate from the first four generations but more persistent over time. Moreover, matrix size and eccentricity stayed constant over time in the salt environment. Changes in the **P**-matrix for salt may thus reveal changes at the underlying genetic level, or may indicate that a decrease in epigenetic effects on the matrix structure after generation 4 was combined with an increase in changes in the **G**-matrix. Although our results did not allow us to rule out these two hypotheses, they may partly explain why we found faster evolutionary rates for salt compared to uranium from generations 4 to 22. A stable matrix implies stronger genetic association between the traits. These results confirm those of a previous study using isogenic lines of the same *C. elegans* population, in which we found a stronger and positive genetic correlation between fertility and growth in salt than in uranium (M. Dutilleul, unpublished observations). Stronger genetic association may constrain the independent evolution of traits [14], but it can also facilitate the evolution of both traits if they are positively correlated and both positively selected.

Our results confirm the hypothesis of matrix instability and provide new evidence that epigenetic effects, selection and genotype by environment interaction can have instantaneous and strong effects on the divergence in the orientation, eccentricity and size of the matrix in a polluted environment.

### Rates of evolution

Pollutants in our study affected the rate of evolution of populations, with a stronger evolutionary response to salt than uranium treatment. There was a 2.2% increase per generation for total fertility in the former, mostly caused by a strong 4.8% increase for late fertility. In addition, generation time has been found to be longer in the salt than in the uranium or control treatments (i.e. around 4 days instead of 3; M. Dutilleul, unpublished observations). Consequently, salt populations were studied for less than 22 generations, and evolutionary responses were probably underestimated.

Why would the evolutionary response be faster to salt than uranium treatment? We chose concentrations that reduced fertility by almost 60% at the first generation for both pollutants. However, despite that precaution, selection pressures could be stronger in salt as suggested by the stronger reduction in survival in this medium [see Additional file 5]. Indeed some genotypes may have been removed faster from the salt population. Heritability of traits in uranium is lower than in the other treatments (M. Dutilleul, unpublished observations), and differences in genetic structures for uranium compared to salt (see above) could also constrain the evolutionary potential of traits in that medium (see e.g. [64]). Populations also showed a stronger acclimation to uranium than to salt during the first generations of exposure [intercepts in Additional file 2 and see Additional file 4]. The quick acclimation may have also reduced the strength of selection on the traits and thus the evolutionary rate in the uranium populations [65]. A population’s response to a novel environment by acclimation or by adaptive processes can have completely different implications on the future of the population. Although plasticity is a costly strategy [66], it does not entail any long-term costs of adaptation such as a reduction of genetic diversity [6,8,67]. Consequently, populations that respond to a novel environment by plasticity can cope with a larger range of conditions.

## Conclusion

We have shown that rapid adaptation to different polluted environments may involve different and complex patterns of evolutionary responses of the life history traits with consequences at the demographic level. Part of this differential response is caused by the shape and the strength of selection pressures on the studied traits, the capacity of populations to acclimate to novel conditions through phenotypic plasticity, some epigenetic effects and the direct effects of the pollutants on the genetic (co)variance structure of traits. Studies on microevolutionary responses to pollutants should thus incorporate information on these different aspects of the response of populations that will help highlight the consequences of pollution on the evolutionary potential of wild populations.

## Methods

### Population maintenance

*C. elegans* is characterized by a short life cycle, small body length and great ease of handling, and is thus a good model for evolution experiments [68]. *C. elegans* experiments do not require approval as specified by general guidelines of the CNRS regarding experimentation using invertebrates. Rather than characterizing the potential evolutionary response of a given *C. elegans* population to novel environmental conditions we were interested in examining the global evolutionary patterns that could occur in response to pollutants. Therefore we chose to work with a stock population composed of a mixture of 16 wild isolates [69] to obtain a large genetic diversity. Prior to our study, the population was kept for > 140 generations in the experimental conditions described in Teotónio *et al.* [69], where recombination–selection equilibrium was mostly achieved without significant loss of genetic diversity. The population was composed of around 30% of males for an androdioecious breeding system (i.e. self-fertilizing hermaphrodites with facultative outcross with males). For our study we changed laboratory conditions: we used 500 individuals in a 9-cm Petri plate with NGM-modified agar (i.e. use of HEPES buffer; for more details see [70]). We produced six replicated experimental populations.

We grew *Escherichia coli* OP50 cultures in Lysogeny Broth (LB) rich medium at 37°C overnight. To avoid interaction between LB and U in the future U-treatment, we systematically centrifuged bacteria twice, removed the supernatant and re-suspended bacteria with a solution of 85 mM NaCl to obtain a 20:1 mixture of *E. coli* (OD_600nm_ of 3 in LB). Plates were seeded with 1 ml of this food source. Then plates were top-exposed to UV doses for 90 s to stop bacterial growth (Bio-Link Crosslinker; λ = 254 nm; intensity = 200 μW.cm^−2^). The main aim of this UV treatment was to avoid differential bacterial growth in control and polluted plates.

Every 3 days we twice washed the nematodes off the plates with 3 ml of M9-modified solution (use of HEPES buffer) for each replicate and mixed them with each other in a 50-ml Falcon tube to ensure we kept a single population. The number of individuals in the tube was counted with five sample drops of 5 μl (see [69]), and then the volume corresponding to 500 individuals, from all developmental stages, was placed in a fresh Petri plate. This was done to transfer a representative sample of the age structure of the population at each time and avoid unintentional selection of some specific life history strategies. Nematodes were cultured throughout the experiment at 20°C and 80% relative humidity.

### Conditions of pollution

After repeating this protocol 40 times (i.e. about 40 generations), the individuals from the six replicates were mixed with each other for the last time before being transferred by groups of 500 individuals in each of the four different conditions (six replicates per condition). We maintained the novel populations in similar conditions to previously, except that replicates were kept separate from each other in 15-ml Falcon tubes. The medium differed according to the four conditions of the experiment: (1) a control environment (see above for medium) and three stressful environments, identical to the control, except for the addition in the NGM-modified agar of (2) 1.1 mM U [uranyl nitrate: UO_2_(NO_3_)_2_ · 6H_2_O; Sigma-Aldrich, France], (3) 308 mM NaCl or (4) alternating uranium-salt for each generation (in the same conditions as for treatments 2 and 3; U for even generations and salt for odd generations). Uranium and salt concentrations were chosen because they reduced fertility by about 60% with the first generation of exposure –corresponding to strong selection. In all media we added 51 mM NaCl as in the classical preparation of NGM [71], except in the salt environment where the concentration was 308 mM NaCl. This multigenerational experience of selection lasted approximately 22 generations (one generation per 3 days). Populations experienced longer generation time in salt, but to simplify we used a generation time of 3 days for all treatments. Hereafter we will refer to the different populations in the different treatments as control, uranium, salt and alternating populations.

### Traits measurements

At any given generation, 3 days after the transfer of individuals from the previous generation in the Petri plate, we transferred 500 individuals from each population onto a new 6-cm Petri plate to build up the next generation. We used the individuals left in the Falcon tubes to estimate the rate of increase of populations (see ‘Estimation of the rate of increase of populations’).

After cleaning individuals off the Petri plate, there were still hundreds of eggs adhering to the plate surface. We used these eggs for trait measures at generations 1–4, 7, 10, 13, 16, 20 and 22. Approximately 100 eggs were taken from the original Petri plate and transferred onto a 6-cm Petri plate containing 10 ml of NGM (same medium as the original Petri plate) and 250 μl of 5:1 UV-killed OP50 (OD_600nm_ of 3). After 48 h, we could determine the sex of individuals, and thus we measured phenotypic traits on both hermaphrodites and males.

To measure brood size, and index of fertility, three hermaphrodites per replicate were transferred individually onto a well of a 12-well tissue culture plate (same medium as the original Petri plate; 2 ml of NGM per well and 75 μl of 5:1 UV-killed OP50). Brood size was measured as the number of hatched progeny produced by a hermaphrodite. We measured brood size before and after 96 h of age to obtain an index of early and late fertility.

We measured morphological traits using pictures of individuals taken with a stereomicroscope (Olympus SZX12, 1.6 × 90 magnification) and a computer-connected camera (Nikon D5000). Males and hermaphrodites were measured at 96 h. We measured male body length using a rapid and automatic image analysis procedure developed in Matlab (R2010b, Mathworks ©). First a background subtraction was applied and the body was extracted by a classic thresholding method. A skeletonization algorithm was then used to obtain the relevant body points, which serve as basis for a spline of interpolation to measure the precise length of each individual [see Additional file 6 for more details on the automatic procedure]. The presence of bacteria in the plate for hermaphrodites (see fertility measure above) prevented us from automatically differentiating individual hermaphrodites. Consequently, we used ImageJ software [72] and measured their body length manually. A strong correlation between automatic and manual measures in a subsample of males (r = 0.97, n = 15) validated the automatic procedure. Body length was used as an index of growth during 0–96 h of age.

We finally measured male body bend frequency at 96 h, as an index of locomotory behavior (measure of the same males as for body length). Movement is important for foraging, microhabitat selection and mating [47,48], and thus any effect of the pollutant on body bend may have serious consequences for fitness. One body bend equals a change in the direction of the anterior part of the worm (including the posterior bulb of the pharynx) along the Y-axis using the body of the worm as the X-axis [73]. Individuals on a 6-cm Petri plate were washed twice with washing buffer that permitted a rapid sedimentation of individuals in the liquid [74]. The buffer was composed of 5 mM HEPES, 1 mM CaCl_2_, 1 mM MgSO_4_ and 0.5 g.l^−1^ gelatin. Individuals were then placed onto a 6-cm Petri plate containing 10 ml of NGM but no bacteria. After 5 min, we counted the number of body bends over 20 s of three males per replicate.

### Estimation of the rate of increase of populations

At each generation after having counted the number of individuals in the five sample drops of 5 μl and transferred the 500 individuals used to found the next generation (see ‘Population maintenance’ and ‘Traits measurements’), we could easily estimate the population size in the total volume of the Falcon tubes. Estimated population size represents the rate of increase of the population within a period of 3 days. Each Petri plate started with 500 individuals; the number of individuals estimated at the end of 3 days depended on survival, fertility and growth rate in the population. Therefore we expect that populations with rapid life-history traits would also show a large population size after 3 days. Furthermore, demographic changes provide information on the potential reduction in genetic variance or increased genetic drift in the populations as a result of harsh selection imposed by environmental conditions [8].

### Effects on average value of traits

We assumed that changes in the average value of the traits during the first four generations could result from either plastic responses (e.g. individual phenotypic plasticity, parental and grand-parental effects) or evolutionary responses to selection. Parental effects on changes across generations can persist over a few generations [38]. Because of the complexity in separating these effects, the representation of the traits in the first generations was only introduced in Additional file 4. In contrast, after generation 4 the changes in the traits across generations caused by individual and trans-generation phenotypic plasticity could be assumed to be negligible (i.e. non-genetic effects remain constant throughout generations), leaving evolutionary changes responsible for the observed phenotypic changes across generations. Analyses of evolutionary (i.e. genetic) changes were thus restricted to data collected between generations 4 and 22.

We used a Bayesian approach and the MCMCglmm package for generalized linear mixed-effects models [75] in the R software [76]. We fitted multivariate generalized linear mixed-effects models on traits measured in hermaphrodites (i.e. total, early and late fertility, and growth) and in males (i.e. body bend and growth). We also used univariate models to analyze temporal changes in population size. For each model we successively added environment, generation (as a continuous variable) and their interactions as fixed effects, and we included replicate populations as a random effect. Estimation of the variance among replicates on the phenotypic changes of traits throughout the study allowed us to test for the occurrence of stochastic effects (i.e. genetic drift) on evolutionary changes. For each selected model we estimated the effects caused by differences between replicates on the total (co)variance by dividing the between-replicate (co)variance by the sum of within- and between-replicate (co)variance. We modeled all traits with a normal error structure. The multivariate analysis allowed us to estimate a full matrix of posterior distributions of (co)variance for all the traits together in the model, and to take into account that associated traits may not evolve independently of each other.

Prior to analysis, we rescaled the traits by subtracting each value by the mean of the sample (i.e. all data for a trait across generations and treatments) and dividing it by twice the standard deviation [77]. After having tested different priors (see e.g. [78]), we retained a proper prior [*nu* = *k* – 1 + 0.002] with a very low variance parameter [*V* = diag(*k*) × *V*_*p*_ × 0.05], where *V*_*p*_ is the phenotypic variance and *k* the dimension of *V* (i.e. number of traits). We allowed models to estimate different random and residual variances, and covariances between pairs of traits. After checking for the convergence of parameter values (i.e. number of iterations, burn-in phase and thinning) and the absence of autocorrelation, we retained 110 000 iterations with a burn-in phase of 10 000, for a total of 1000 samples for each analysis.

We compared DIC of models including different effects. We retained, as the best-fitted model, the model with the lowest DIC and this DIC differed from the second best-fitted model’s DIC by > 5 [79]. When two models had DICs within a range of 5, we retained the most parsimonious model (i.e. with the lowest number of parameters).

For each trait we previously fitted univariate models with temporal autocorrelation using the nlme package [80]. We wanted to check if the significant differences were the same as for our multivariate models without correction for temporal dependency. Although the *p*-values (nlme) slightly differed from *p*-MCMC (MCMCglmm), the significant effects (i.e. *p*-MCMC ≈ *p*-value < 0.05) were the same in both models (data not shown). *p*-MCMC is the proportion of cases in which the samples from the MCMC chains is less than the significance level (here 0.05), equivalent to *p*-values [75,78].

We used the posterior distribution of each trait analyzed to estimate the parameter value and the limit of the 95% HPDI. We considered significant differences for a trait between two environments or two different generations, when the 95% HPDI for the subtraction between the whole posterior distributions of both estimates did not overlap 0. We used ‘significant’ even if with a Bayesian approach significance reflects more a difference that is considered non-negligible (i.e. it differs from the significance level commonly used in a frequentist approach). To estimate evolutionary responses to selection, we used the posterior mode of the distribution of both the intercept (i.e. an estimate of the relative level of the trait for each treatment at generation 4), and the slope of the linear regression of each treatment as a function of generations (i.e. an estimate of the between-generation change in the trait in one treatment relative to the control).

### Effects on the matrices of trait (co)variance

We assessed the divergence between the matrices of trait (co)variance at different periods of time and between treatments. The phenotypic (co)variance matrix (i.e. **P**-matrix) does not always reflect the structure of the **G**-matrix [81]. We assumed that the matrix we estimated between generations 1 and 4 included both genetic and non-genetic effects (i.e. phenotypic plasticity, parental and grand-parental effects), and thus represented the **P**-matrix. However, in our experiment treatment conditions were kept constant from generation 1 onward and, by generation 4, changes caused by within- and cross-generation phenotypic plasticity [82,83] were assumed to be negligible. Therefore, after generation 4, changes in the matrix of trait (co)variance essentially reflected changes in the **G**-matrix.

For each of the three constant environments (i.e. control, uranium and salt), we estimated one matrix for traits measured in hermaphrodites (i.e. total, early and late fertility, and growth) and one for traits measured in males (i.e. body bend and growth). Instability of the **G**-matrix in the alternative treatment prevented us comparing it with the other treatments. We thus chose not to present results for that treatment. To increase the robustness of analyses, we assessed the temporal changes in each treatment by pooling data over successive generations (i.e. data for generations 1–4, 4–10 and 13–22); these pooled data were used to estimate the three successive matrices **G**_1_, **G**_2_ and **G**_3_, respectively. We also estimated a combined matrix **G**_4_ (generations 4–22). For each treatment we ran pairwise comparisons between each successive matrix (**G**_1_ vs. **G**_2_ and **G**_2_ vs. **G**_3_). We also compared matrices for different treatments at the same period (e.g. **G**_1,salt_ vs. **G**_1,uranium_ and **G**_4,salt_ vs. **G**_4,uranium_).

It is now possible to compare all the dimensions of **G**-matrices [81,84]. The difference between the first eigenvector of two matrices explains most of the variation in the orientation of the matrix [15,61]. We combined Bayesian linear mixed-effect models with a bootstrap procedure (resampling of all the individuals with replacement) with 1000 iterations to calculate the angle between the first eigenvector of each matrix. We ran models without any fixed effect and including replicate populations as a random effect. After several tests, we decided to keep the same priors, number of iterations, burn-in phase and thinning as in the models used to analyze the evolutionary responses of traits. We ran a principal component analysis of the obtained matrices to extract their first eigenvector. We then compared matrices two-by-two by calculating the cosine of the angle *θ*, between their first eigenvector, following:

1 $$ \cos \left(\theta \right)=\frac{u_1.{u}_2}{\left\Vert {u}_1\right\Vert .\left\Vert {u}_2\right\Vert }, $$

where *u*_*i*_ and ‖*u*_*i*_‖ are respectively the first eigenvector and the norm of the first eigenvector of matrix i. For each matrix, we also estimated matrix eccentricity and size. Eccentricity is the ratio of the first eigenvalue to the sum of the remaining eigenvalues, and represents the shape of the matrix, a high eccentricity reflecting an elongated, cigar-shaped matrix [14]. Matrix size is defined here as the sum of the eigenvalues and reflects the level of its overall variance and covariance [14].

The bootstrap procedure enabled us to calculate for each angle, eccentricity and size the posterior mode of distribution and the 95% confidence interval (CI). We considered that two estimates were significantly different when the 95% CI of subtraction between their distributions did not overlap 0. In the bootstrap procedure, we automatically corrected the arbitrary change in the sign of the eigenvectors of any particular axis (axis reflection) and the reordering of axes due to very similar eigenvalues.

## Availability of supporting data

The data sets supporting the results of this article are available in the doi:10.5061/dryad.st57b repository [85], [http://datadryad.org/review?wfID=34660&token=1fa5838f-7cc2-4dea-bc1e-0ecd4159a03d].

## Additional files

Additional file 1:
**Analyses of covariance in bivariate models.** The table shows the effect of covariance between hermaphrodite traits (i.e. growth and total, early and late fertility) for all combinations of two traits, measured between generations 4 and 22 of the multigenerational experiment. We used bivariate mixed models with traits included as dependent variables, and compared models with covariance, allowed (Y) or not allowed (N) in the priors, using deviance information criterion (DIC). The associated change (Δ) in DIC between the bivariate models corresponds to the difference between the DIC of the models, including or not the covariance. We retained, as the best-fitted model, the model with the lowest DIC. All the models included replicates as a random effect to control for dependence of data across generations within each replicate, and environment (control, uranium, salt and alternating uranium-salt) and generation as fixed effects. Additional file 2:
**Analyses of the differences between trait values in** ***C. elegans*** **between generations 4 and 22.** The table shows the intercept corresponds to the rescaled traits value at generation 4 and slope corresponds to the slope of linear regressions across generations. Results are shown for hermaphrodites and males. Values correspond to the estimation given by the posterior mode of the distribution for each parameter (i.e. intercept and slope) in control (first line for each parameter) or for each parameter in each environment relative to the others. Traits values were rescaled prior to analysis by subtracting each value from the mean of the sample and dividing it by twice the standard deviation, thus values for the intercepts and slope were measured in the rescale unit and in the rescale unit per generation, respectively. Values between brackets correspond to the limit of the 95% highest posterior density interval (HPDI). Values in bold are those for which the 95% HPDI did not overlap 0. Additional file 3:
**Analyses of population size.** The table shows the effect of generation and environment (control, uranium, salt and alternating uranium-salt) on population size measured between generations 1 and 22 of the multigenerational experiment. (A) We used multivariate mixed models with all the traits included as dependent variables, and compared different models using deviance information criterion (DIC). All the models included replicates as a random effect to control for dependence of data across generations within each replicate. The first DIC value corresponds to a simple model including only replicates as a random effect. The next values corresponding to the DIC of the next model included a given fixed effect and the associated change (Δ) in DIC between the two models including or not including that fixed effect. In bold, models for which ΔDIC > 5, i.e. the model including interaction had a smaller DIC, for which the replicate effect was 6.2%. (B) Analyses of differences for population size. Intercept corresponds to the population size at the generation 1 and slope corresponds to the slope of linear regressions across generations. Values correspond to the estimation given by the posterior mode of the distribution for each parameter (i.e. intercept and slope) in control (first line) or for each parameter in each environment relative to the others. Values between brackets correspond to the limit of the 95% highest posterior density interval (HPDI). Values in bold are those for which the 95% HPDI did not overlap 0. Additional file 4:
**Measures of traits in the first four generations.** The figures show the phenotypic response of hermaphrodite total fertility (A), growth (B), early (C) and late (D) fertility and male growth (E) and body bend (F) between generations 1 and 4. Each symbol corresponds to the mean value of the trait and its standard error on 18 individuals at one particular generation. Control (empty triangle), uranium (filled black dots), salt (empty dots) and alternating uranium-salt (filled gray dots) environments. Additional file 5:
**Percentage of survival until 48 h and ratio of males.** The figures show the changes in average survival (A) and sex ratio (B) in the different treatments, between generations 1 and 22. Symbols show the mean value and standard error over six replicated populations in control (empty triangle), uranium (filled black dots), salt (empty dots) and alternating uranium-salt (filled gray dots) treatments. Additional file 6:
**Automatic procedure to measure body length.** The figures show the automatic procedure to measure body length of *C. elegans.* Given an individual body picture (A), the algorithm first applies a background subtraction and a thresholding process in order to differentiate the body from the background (B, C). Then a skeletonization of the body is done (D), and the relevant points on the skeleton are kept (E) to form the basis of the interpolating spline whose length gives the precise measure of the body (F) after correction. This is done regarding the characteristics of the machine in charge of the acquisition process which are: the objective size (OBJ), the captor horizontal width (CAP) in mm, the number of pixels horizontally on the captor (PIX) and finally the transfer function (TRANSF). Then, a coefficient of correction is calculated: coeff = CAP / PIX / OBJ / TRANSF. The distance in pixel is multiplied by this coefficient in order to obtain the real distance in mm.
