# Supplementary material for: Rapid evolutionary responses of life history traits to different experimentally-induced pollutions in Caenorhabditis elegans
Source: BMC Evol Biol. 2014 Dec 10;14:252. doi: 10.1186/s12862-014-0252-6 (PMC4272515; doi:10.1186/s12862-014-0252-6)
Supplement: Additional file 1: — Analyses of covariance in bivariate models. The table shows the effect of covariance between hermaphrodite traits (i.e. growth and total, early and late fertility) for all combinations of two traits, measured between generations 4 and 22 of the multigenerational experiment. We used bivariate mixed models with traits included as dependent variables, and compared models with covariance, allowed (Y) or not allowed (N) in the priors, using deviance information criterion (DIC). The associated change (Δ) in DIC between the bivariate models corresponds to the difference between the DIC of the models, including or not the covariance. We retained, as the best-fitted model, the model with the lowest DIC. All the models included replicates as a random effect to control for dependence of data across generations within each replicate, and environment (control, uranium, salt and alternating uranium-salt) and generation as fixed effects. [file 12862_2014_252_MOESM1_ESM.doc]

**Additional file 1. Analyses of covariance in bivariate models.**

The table shows the effect of covariance between hermaphrodite traits (i.e. growth and total, early and late fertility) for all combinations of two traits, measured between generations 4 and 22 of the multigenerational experiment. We used bivariate mixed models with traits included as dependent variables, and compared models with covariance, allowed (Y) or not allowed (N) in the priors, using deviance information criterion (DIC). The associated change (Δ) in DIC between the bivariate models corresponds to the difference between the DIC of the models, including or not the covariance. We retained, as the best-fitted model, the model with the lowest DIC. All the models included replicates as a random effect to control for dependence of data across generations within each replicate, and environment (control, uranium, salt and alternating uranium-salt) and generation as fixed effects.
