# Supplementary material for: Rapid evolutionary responses of life history traits to different experimentally-induced pollutions in Caenorhabditis elegans
Source: BMC Evol Biol. 2014 Dec 10;14:252. doi: 10.1186/s12862-014-0252-6 (PMC4272515; doi:10.1186/s12862-014-0252-6)
Supplement: Additional file 2: — Analyses of the differences between trait values in C. elegans between generations 4 and 22. The table shows the intercept corresponds to the rescaled traits value at generation 4 and slope corresponds to the slope of linear regressions across generations. Results are shown for hermaphrodites and males. Values correspond to the estimation given by the posterior mode of the distribution for each parameter (i.e. intercept and slope) in control (first line for each parameter) or for each parameter in each environment relative to the others. Traits values were rescaled prior to analysis by subtracting each value from the mean of the sample and dividing it by twice the standard deviation, thus values for the intercepts and slope were measured in the rescale unit and in the rescale unit per generation, respectively. Values between brackets correspond to the limit of the 95% highest posterior density interval (HPDI). Values in bold are those for which the 95% HPDI did not overlap 0. [file 12862_2014_252_MOESM2_ESM.doc]

**Additional file 2. Analyses of the differences between trait values in *C. elegans* between generations 4 and 22.**

The table shows the intercept corresponds to the rescaled traits value at generation 4 and slope corresponds to the slope of linear regressions across generations. Results are shown for hermaphrodites and males. Values correspond to the estimation given by the posterior mode of the distribution for each parameter (i.e. intercept and slope) in control (first line for each parameter) or for each parameter in each environment relative to the others. Traits values were rescaled prior to analysis by subtracting each value from the mean of the sample and dividing it by twice the standard deviation, thus values for the intercepts and slope were measured in the rescale unit and in the rescale unit per generation, respectively. Values between brackets correspond to the limit of the 95% highest posterior density interval (HPDI). Values in bold are those for which the 95% HPDI did not overlap 0.
