# Supplementary material for: Rapid evolutionary responses of life history traits to different experimentally-induced pollutions in Caenorhabditis elegans
Source: BMC Evol Biol. 2014 Dec 10;14:252. doi: 10.1186/s12862-014-0252-6 (PMC4272515; doi:10.1186/s12862-014-0252-6)
Supplement: Additional file 3: — Analyses of population size. The table shows the effect of generation and environment (control, uranium, salt and alternating uranium-salt) on population size measured between generations 1 and 22 of the multigenerational experiment. (A) We used multivariate mixed models with all the traits included as dependent variables, and compared different models using deviance information criterion (DIC). All the models included replicates as a random effect to control for dependence of data across generations within each replicate. The first DIC value corresponds to a simple model including only replicates as a random effect. The next values corresponding to the DIC of the next model included a given fixed effect and the associated change (Δ) in DIC between the two models including or not including that fixed effect. In bold, models for which ΔDIC > 5, i.e. the model including interaction had a smaller DIC, for which the replicate effect was 6.2%. (B) Analyses of differences for population size. Intercept corresponds to the population size at the generation 1 and slope corresponds to the slope of linear regressions across generations. Values correspond to the estimation given by the posterior mode of the distribution for each parameter (i.e. intercept and slope) in control (first line) or for each parameter in each environment relative to the others. Values between brackets correspond to the limit of the 95% highest posterior density interval (HPDI). Values in bold are those for which the 95% HPDI did not overlap 0. [file 12862_2014_252_MOESM3_ESM.doc]

**Additional file 3. Analyses of population size.**

The table shows the effect of generation and environment (control, uranium, salt and alternating uranium-salt) on population size measured between generations 1 and 22 of the multigenerational experiment. (A) We used multivariate mixed models with all the traits included as dependent variables, and compared different models using deviance information criterion (DIC). All the models included replicates as a random effect to control for dependence of data across generations within each replicate. The first DIC value corresponds to a simple model including only replicates as a random effect. The next values corresponding to the DIC of the next model included a given fixed effect and the associated change (Δ) in DIC between the two models including or not including that fixed effect. In bold, models for which ΔDIC > 5, i.e. the model including interaction had a smaller DIC, for which the replicate effect was 6.2%. (B) Analyses of differences for population size. Intercept corresponds to the population size at the generation 1 and slope corresponds to the slope of linear regressions across generations. Values correspond to the estimation given by the posterior mode of the distribution for each parameter (i.e. intercept and slope) in control (first line) or for each parameter in each environment relative to the others. Values between brackets correspond to the limit of the 95% highest posterior density interval (HPDI). Values in bold are those for which the 95% HPDI did not overlap 0.
