# Supplementary material for: Rapid evolutionary responses of life history traits to different experimentally-induced pollutions in Caenorhabditis elegans
Source: BMC Evol Biol. 2014 Dec 10;14:252. doi: 10.1186/s12862-014-0252-6 (PMC4272515; doi:10.1186/s12862-014-0252-6)
Supplement: Additional file 4: — Measures of traits in the first four generations. The figures show the phenotypic response of hermaphrodite total fertility (A), growth (B), early (C) and late (D) fertility and male growth (E) and body bend (F) between generations 1 and 4. Each symbol corresponds to the mean value of the trait and its standard error on 18 individuals at one particular generation. Control (empty triangle), uranium (filled black dots), salt (empty dots) and alternating uranium-salt (filled gray dots) environments. [file 12862_2014_252_MOESM4_ESM.doc]

**Additional file 4. Measures of traits in the first four generations.**

The figures show the phenotypic response of hermaphrodite total fertility (A), growth (B), early (C) and late (D) fertility and male growth (E) and body bend (F) between generations 1 and 4. Each symbol corresponds to the mean value of the trait and its standard error on 18 individuals at one particular generation. Control (empty triangle), uranium (filled black dots), salt (empty dots) and alternating uranium-salt (filled gray dots) environments.
