# Supplementary material for: Rapid evolutionary responses of life history traits to different experimentally-induced pollutions in Caenorhabditis elegans
Source: BMC Evol Biol. 2014 Dec 10;14:252. doi: 10.1186/s12862-014-0252-6 (PMC4272515; doi:10.1186/s12862-014-0252-6)
Supplement: Additional file 5: — Percentage of survival until 48 h and ratio of males. The figures show the changes in average survival (A) and sex ratio (B) in the different treatments, between generations 1 and 22. Symbols show the mean value and standard error over six replicated populations in control (empty triangle), uranium (filled black dots), salt (empty dots) and alternating uranium-salt (filled gray dots) treatments. [file 12862_2014_252_MOESM5_ESM.doc]

**Additional file 5. Percentage of survival until 48 h and ratio of males.**

The figures show the changes in average survival (A) and sex ratio (B) in the different treatments, between generations 1 and 22. Symbols show the mean value and standard error over six replicated populations in control (empty triangle), uranium (filled black dots), salt (empty dots) and alternating uranium-salt (filled gray dots) treatments.
