# Supplementary material for: Rapid evolutionary responses of life history traits to different experimentally-induced pollutions in Caenorhabditis elegans
Source: BMC Evol Biol. 2014 Dec 10;14:252. doi: 10.1186/s12862-014-0252-6 (PMC4272515; doi:10.1186/s12862-014-0252-6)
Supplement: Additional file 6: — Automatic procedure to measure body length. The figures show the automatic procedure to measure body length of C. elegans. Given an individual body picture (A), the algorithm first applies a background subtraction and a thresholding process in order to differentiate the body from the background (B, C). Then a skeletonization of the body is done (D), and the relevant points on the skeleton are kept (E) to form the basis of the interpolating spline whose length gives the precise measure of the body (F) after correction. This is done regarding the characteristics of the machine in charge of the acquisition process which are: the objective size (OBJ), the captor horizontal width (CAP) in mm, the number of pixels horizontally on the captor (PIX) and finally the transfer function (TRANSF). Then, a coefficient of correction is calculated: coeff = CAP / PIX / OBJ / TRANSF. The distance in pixel is multiplied by this coefficient in order to obtain the real distance in mm. [file 12862_2014_252_MOESM6_ESM.doc]

**Additional file 6. Automatic procedure to measure body length.**


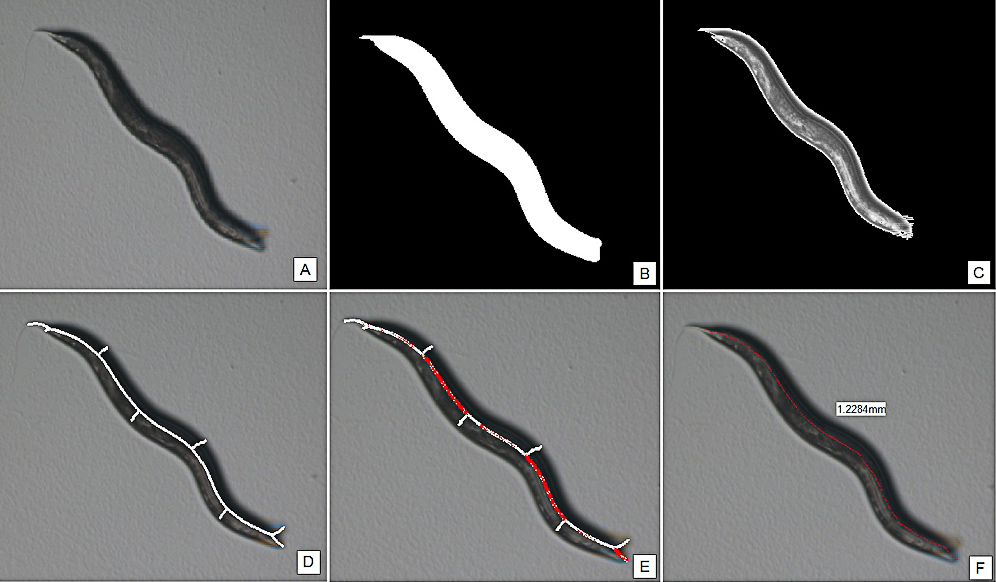


The figures show the automatic procedure to measure body length of *C. elegans.* Given an individual body picture (A), the algorithm first applies a background subtraction and a thresholding process in order to differentiate the body from the background (B, C). Then a skeletonization of the body is done (D), and the relevant points on the skeleton are kept (E) to form the basis of the interpolating spline whose length gives the precise measure of the body (F) after correction. This is done regarding the characteristics of the machine in charge of the acquisition process which are: the objective size (OBJ), the captor horizontal width (CAP) in mm, the number of pixels horizontally on the captor (PIX) and finally the transfer function (TRANSF). Then, a coefficient of correction is calculated: coeff = CAP / PIX / OBJ / TRANSF. The distance in pixel is multiplied by this coefficient in order to obtain the real distance in mm.
